# Assessing the impact of micro and nanoplastics on the productivity of vegetable crops in terrestrial horticulture: a comprehensive review

**DOI:** 10.1007/s10661-025-13820-1

**Published:** 2025-03-17

**Authors:** Harshana Galahitigama, Poorni Sandamali, Thilini Jayapra, Nandula Abesinghe, Mudalige Don Hiranya Jayasanka Senavirathna, Ma Brida Lea Diola, Maria Antonia Tanchuling

**Affiliations:** 1 Graduate School of Science and Engineering, Saitama University, 255 Shimo-Okubo, Sakura-Ku, Saitama, 338-8570 Japan; 2 Faculty of Agricultural Sciences, Sabaragamuwa University of Sri Lanka, P.O. Box 02, Belihuloya, 70140 Sri Lanka; 3 Department of Agricultural Technology, Faculty of Technology, University of Colombo, Pitipana, Homagama, Sri Lanka; 4 Institute of Civil Engineering, College of Engineering, University of the Philippines Diliman, Quezon City, Philippines

**Keywords:** Micro and nano plastics, Plant toxicity, Remediation strategies, Soil properties, Vegetable plants

## Abstract

Micro and nano plastics (MNPs) pollution has emerged as a significant environmental issue in recent years. Plastic contamination in the environment poses risks to both human health and other organisms within the ecosystem. This review discusses the overall impact of MNPs on the performance of vegetable crops, including a global perspective on the topic. Bibliometric analysis reveals that most research on this subject has been concentrated in a few countries, although the number of studies has notably increased in recent years. MNPs accumulate in arable lands due to human activities, often altering the soil's physical, chemical, and biological properties in the rhizosphere. Vegetable crops absorb these MNPs mainly through their roots, leading to accumulation in the edible parts of the plants. Consequently, this results in phytotoxic symptoms and poor growth and development. The phytotoxic effects of MNPs are attributed to genetic and metabolic changes within the plant's cellular structure. Current research on MNPs has been limited to a few vegetable cultivars. Future studies should encompass a broader range of vegetable crops under both laboratory and field conditions to advance this burgeoning field of research. Additionally, examining various types of plastics is essential to comprehensively understanding their impact.

**Graphical Abstract:**

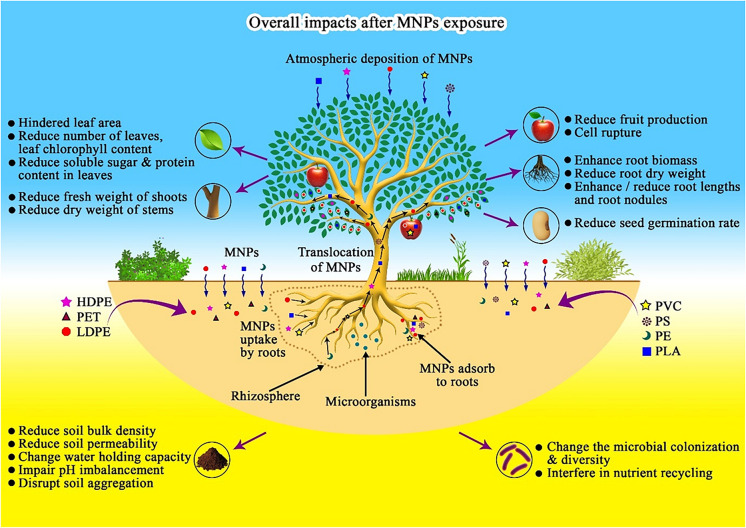

## Introduction

With the growing population, people are increasingly using numerous plastic products to make their lives more convenient. According to recent literature, global plastic production was approximately 390.7 million tons in 2021 (Mamun et al., 2023). Both developed and developing countries have produced more plastic products over the past decade (Gan et al., 2023). Although environmental plastic pollution remains a significant challenge, every nation is making reasonable efforts to mitigate plastic accumulation in the ecosystem. Nevertheless, approximately 60% of plastics remain in the environment (Mamun et al., 2023).

When micro and nano plastics (MNPs) accumulate, they alter the soil's physicochemical and biological properties (De Souza Machado et al., 2018). MNPs directly affect soil nutrient dynamics, often causing imbalances in carbon and nitrogen levels (Torres et al., 2021). Additionally, MNPs can alter the soil microbial community, impacting soil biochemical processes and changing microbial functional properties, which leads to poor organic matter decomposition (Zantis et al., 2023). Recent studies show that soil-accumulated MNPs enter crops through the roots, translocate to the shoot parts, and cause phytotoxic symptoms, resulting in poor plant health (Yu et al., 2021). These toxic effects are primarily due to reactive oxygen species (ROS)-induced oxidative stress (Qiu et al., 2022). ROS molecules exacerbate lipid peroxidation, protein oxidation, and DNA damage in the cytosol of plant cells, leading to cell death (Gao et al., 2019). Moreover, absorbed MNPs accumulate in the edible parts of crops, such as stems, leaves, flowers, and seeds. Additionally, MNPs act as carriers for various other pollutants, including heavy metals, pesticides, and pathogens, increasing their toxic potential (Sewwandi et al., 2023). Finally, bioaccumulated MNPs enter the food chain, gradually concentrating in the human body and posing health hazards (Lehner et al., 2019).

Although many articles on MNPs have been published, few researchers have studied their impact on vegetables. To ensure a healthy diet, the EAT-Lancet Commission recently recommended consuming 300 g per capita per day of vegetables. Most guidelines, including those from the World Health Organization (WHO), recommend consuming more than 400 g of fruits and vegetables per day, with at least 240 g per capita (Herforth et al., 2019). Furthermore, a plant-based diet that includes vegetables is resource- and environment-efficient throughout its production process, supporting the United Nations Sustainable Development Goals (Willett et al., 2019). Thus, when vegetables are contaminated with MNPs, there is a high potential for health issues in humans. In this review, we focus on the impact of MNPs on the growth and development of vegetable crops. The paper includes a bibliometric analysis and a descriptive analysis of MNPs' effects on vegetable crop performance. Additionally, this review aims to elucidate a) the potential sources of MNP contamination b) how MNPs alter soil physicochemical properties in arable lands c) how MNP contaminants interact with vegetable crops, including their absorption and transport mechanisms d) the diverse impacts, including phenotypic and genetic changes, that occur upon MNP exposure e) the effects of MNPs on the crop rhizosphere, and f) several remediation strategies to mitigate MNP accumulation in vegetables. Through this review, we aim to synthesize existing research findings on the effects of MNPs on vegetable crop growth and development.

## Bibliometric analysis

With the growing focus on the impacts of MNPs in the agricultural sector, it is important to collate and synthesize findings from existing studies to understand the field's intellectual framework. Due to the dynamic nature of this area, regular structured reviews are essential to encapsulate evolving research and identify areas that require further investigation. Despite the availability of various studies addressing the effects of MNPs on soil-based crops, a gap remains for an extensive bibliometric-based review that comprehensively maps the current knowledge landscape. To address this gap, this paper undertakes a bibliometric analysis focusing on the impact of MNPs on vegetable crops. The analysis encompasses scholarly work published from 2000 to 2024, aiming to highlight key studies, categorize the body of literature, and dissect the intellectual framework through co-citation analysis. This approach enables researchers to achieve several objectives, including recognizing essential studies on MNPs in agriculture, delineating the core intellectual structure through semantic analysis of co-citations, and tracing the evolution of keyword and citation networks over time.

**Fig. 1:**
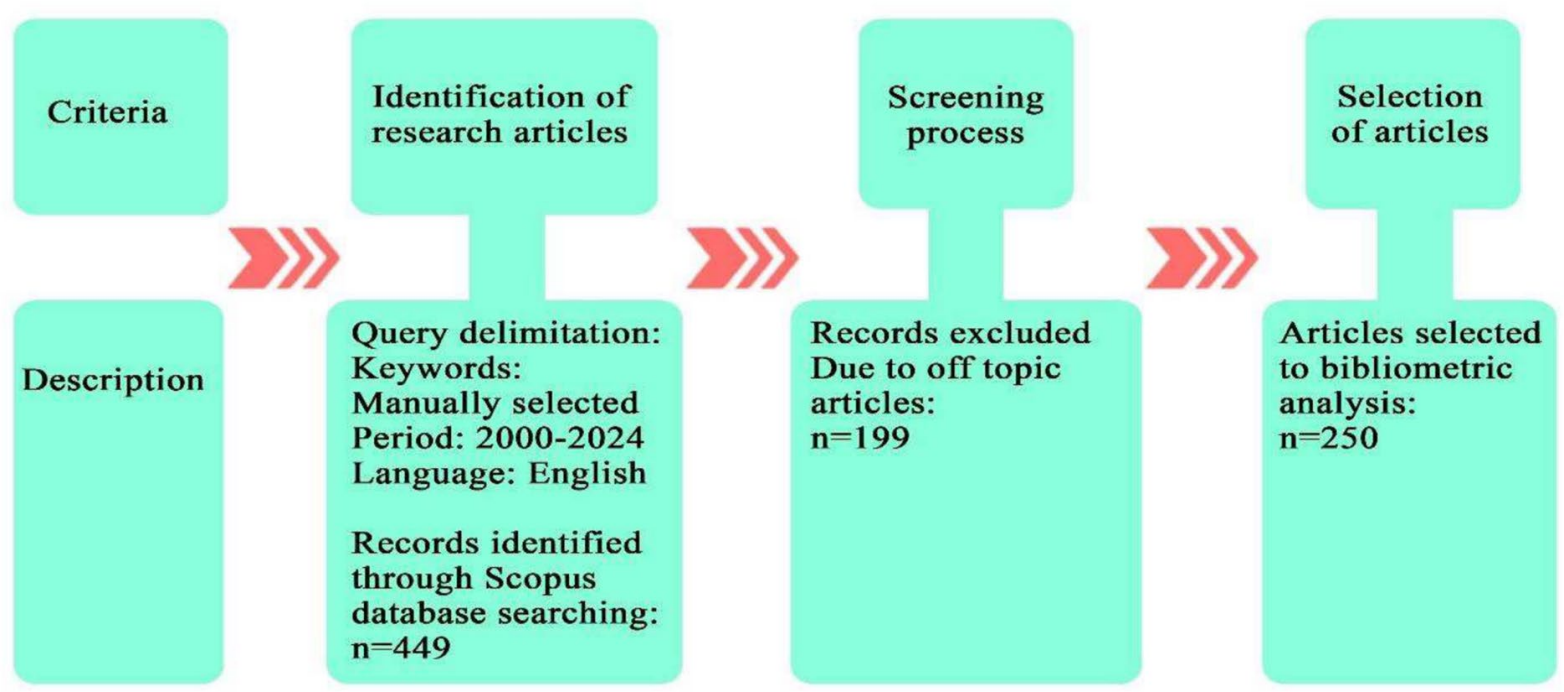
Methodological design of the bibliometric analysis

This analysis aims to comprehensively review how MNPs affect crops by employing bibliometric scrutiny of scientific papers listed in the Scopus database, focusing on geographical, authorship, journal, and keyword aspects. Bibliometric analysis, which leverages statistical and mathematical techniques to review and interpret patterns in scholarly communication and the development of a field (as outlined by pioneers such as Tahai & Rigsby, 1998; Small, 1999), serves as a tool to uncover and understand prevailing themes and contributions to the knowledge domain, according to Thelwall (2008). The bibliometric study followed a three-stage process guided by the PRISMA (Preferred Reporting Items for Systematic Reviews and Meta-Analyses) framework, which outlines essential steps for conducting systematic reviews of scientific studies indexed in bibliographic databases, including the removal of irrelevant articles (Fig. 1). Initially, a search was conducted in the Scopus bibliographic database to create the dataset. The search query included a combination of keywords: ("*microplastic*" *OR* "*nanoplastic*") *AND* ("*terrestrial horticultural crops*" *OR* "*oxidative response in plants*" *OR* "*morphological changes*" *OR* "*uptake and translocation in plants*" *OR* "*metabolic changes in plants*" *OR* "*rhizosphere*" *OR* "*microsphere*" *OR* "*remedial measures*" *OR* "*contamination in agricultural fields*"). The selection criteria required that articles contain at least two of these terms in their title, abstract, or keywords. This search was narrowed to publications from the last 24 years (2000 to June 2024) and was restricted to those published in English, identifying 449 articles in the initial phase.

The second phase involved filtering these articles to remove those unrelated to our research theme, reducing the number to 250 papers, primarily excluding those focused on medical, zoological, and engineering topics. Ultimately, 250 articles were selected to construct the database, encompassing metadata such as authors, journal, country, citation count, and keywords. This collection included original research articles (227), review papers (13), notes (4), conference papers (2), book chapters (2), an editorial (1), and a letter (1).

The tool VOSviewer (VOSviewer, version 1.6.16, Centre for Science and Technology Studies, Leiden University, The Netherlands) was used to visualize results and create bibliometric networks, known for its user-friendly interface for bibliometric mapping. This bibliometric analysis elucidated research trends and prominent topics, detailing contributions and citations, including yearly publication volume, authors, affiliations, countries, journals, most-cited papers, and journals among the publications. We analyzed journal articles to encompass the entire research domain concerning the effect of MNPs on terrestrial horticultural crops.

### Annual publications

**Fig. 2:**
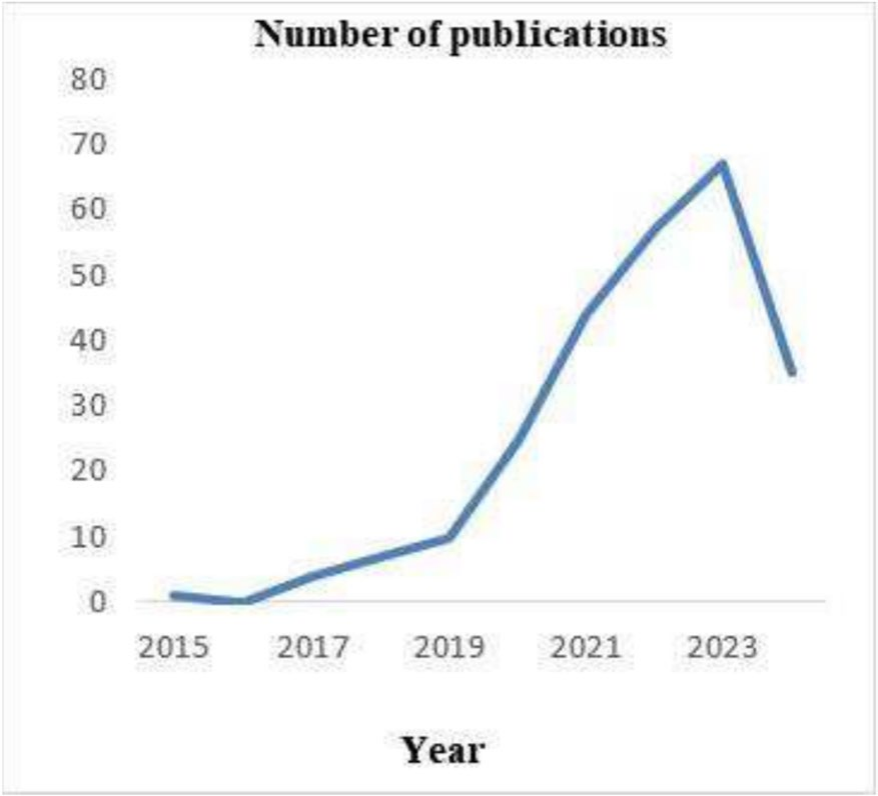
Annual distribution of publications

Initially, we examined the progression of published works in agricultural drones, with the chronological spread of academic research depicted in Fig. 2. Publications began to increase notably in 2017. The number of published articles increased from 10 in 2018 to 25 in 2019, reaching a peak of 67 in 2023. Between 2018 and 2023, a cumulative total of 210 articles were published.

### Leading countries in research conducting

**Fig. 3:**
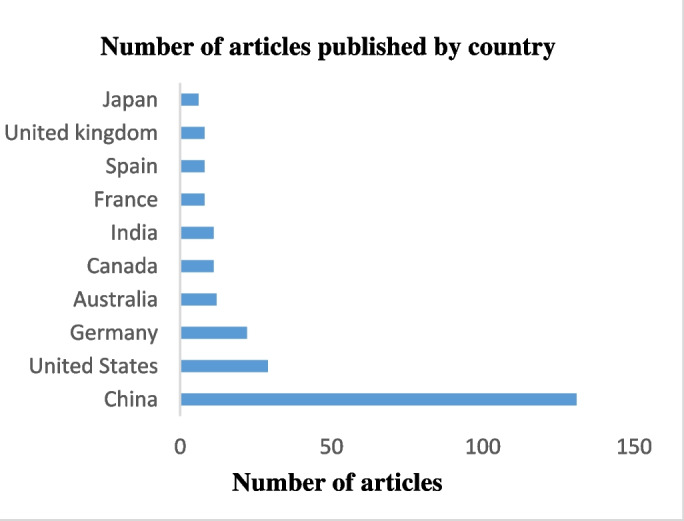
Top most productive countries contributing to relevant publications

Over the past decade, 55 countries have conducted research into the effects of MNPs on agriculture. China, the United States of America, and Germany have emerged as the leading contributors, accounting for 46%, 10%, and 8% of total publications, respectively. Following closely behind these leaders, Australia, Canada, and India have also made significant contributions to the body of research in this field (Fig. 3).

### Most relevant keywords

**Fig. 4:**
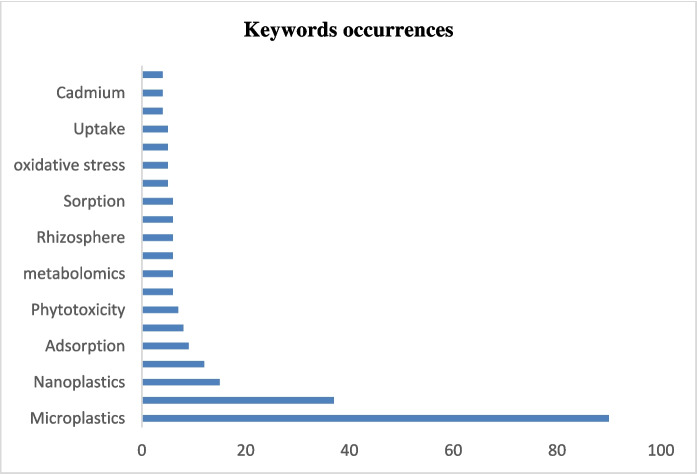
Keyword occurrences in research articles

**Fig. 5:**
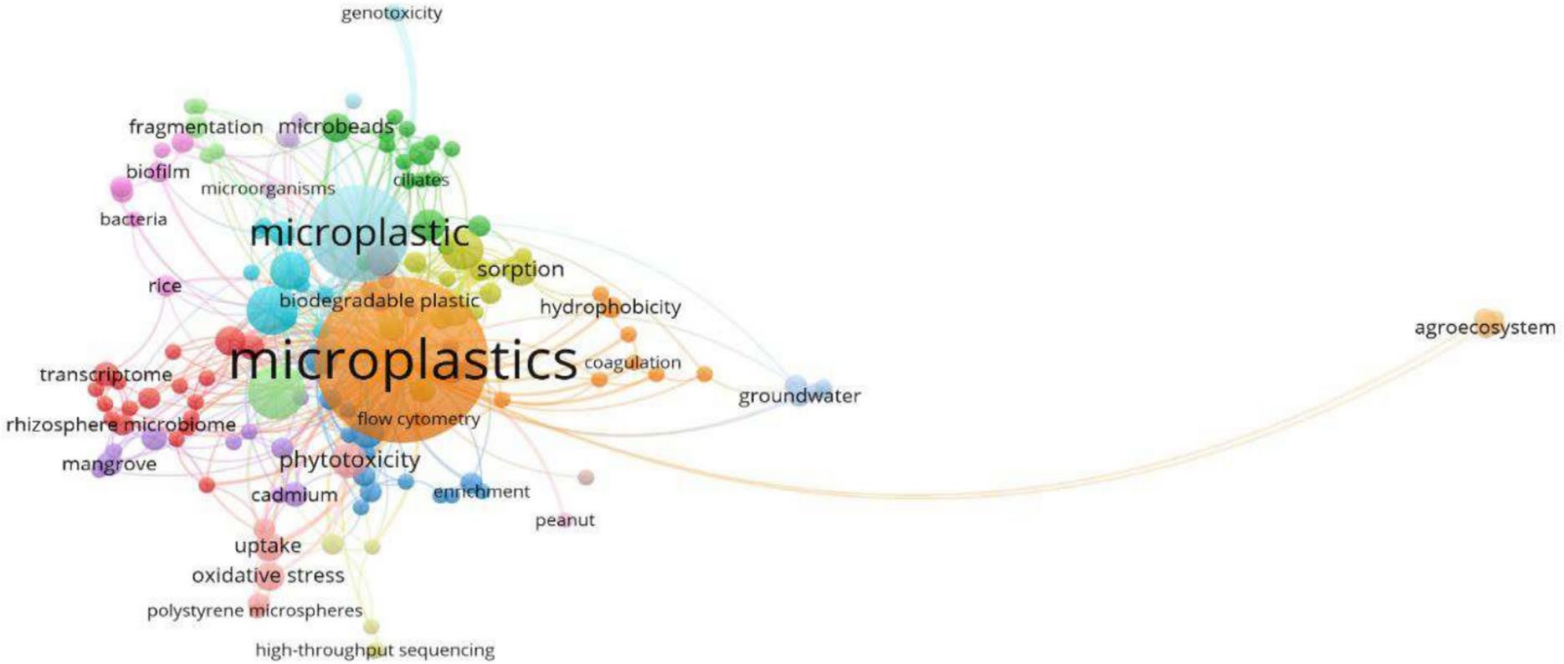
Keyword co-occurrence networks

In the collection of 250 articles, over 791 author keywords were pinpointed. These keywords highlight the topics explored in the last ten years, including microplastic, nanoplastic, polystyrene, adsorption, polyethylene, and phytotoxicity (Fig. 4). Figure 5 illustrates the grouping of keywords that appear most frequently, revealing 18 clusters of author keywords. The most prominent term is "microplastics," indicating it is the central topic of the visualization. Several interconnected terms surrounding "microplastics" suggest research areas or topics such as environmental impact and interactions, material and chemical properties, health and biological effects, etc. Their distance from one another indicates the degree of association between terms in this instance. Terms like "biodegradable plastic," "phytotoxicity," and "biofilm," which are often used in conjunction with "microplastics," are closely grouped (Fig. 5). However, "agroecosystem" is further distant since it might not be directly associated with "microplastics" as much in the dataset. This implies a weaker or more particular association between "agroecosystem" and microplastics, even if it is less commonly used in the same context as other terminologies. The orange lines, however, indicate that "agroecosystem" and other closely connected environmental concepts, such as "sorption" or "groundwater," nonetheless share a conceptual or thematic connection.

## MNP sources and contents in the environment

**Fig. 6:**
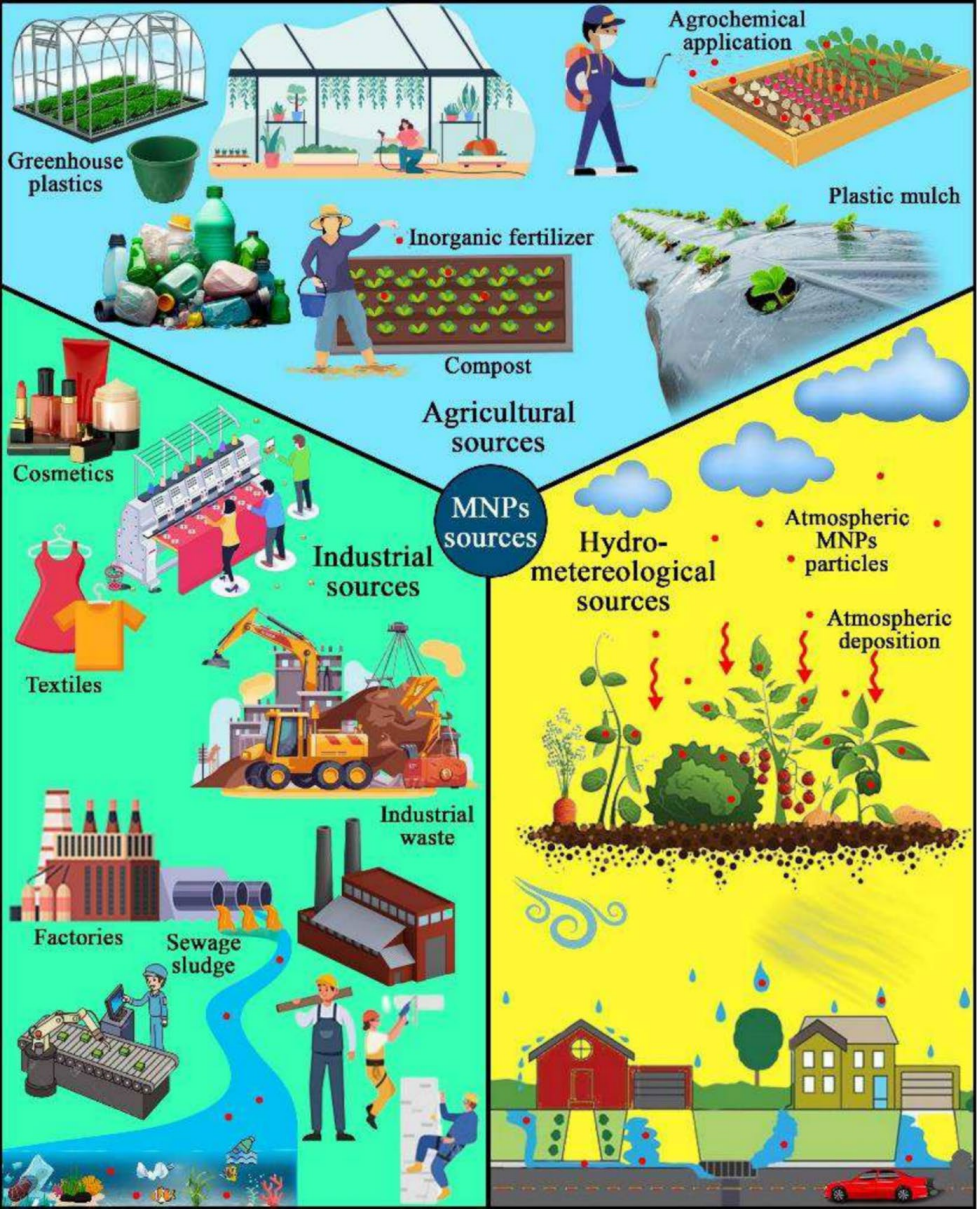
Numerous sources of MNPs in vegetable cultivation. The schematic diagram shows possible pathways to introduce MNPs in vegetable crops, mainly through industrial, agricultural, and hydro-meteorological sources

The sources of MNPs in agricultural fields can be categorized mainly into primary or direct sources, which introduce MNPs directly into the fields, and secondary or indirect sources, which result from the degradation of larger materials (Junhao et al., 2021; Lwanga et al., 2022). Moeck et al. (2022) further classify these sources based on their impacts, including those caused by agricultural practices, urban influences, and hydro-meteorological factors. Overall, potential sources of MNPs in agricultural environments include inputs from the application of sewage sludge, compost, irrigation with wastewater, road runoff, atmospheric deposition, and plastics used in agricultural practices, such as mulch film and greenhouse plastics (Kim et al., 2021; Maddela et al., 2023; Fig. 6).

Agricultural plastic usage significantly contributes to soil microplastics (MPs) (Grause et al., 2022). Plastic mulch, used to suppress weeds and retain soil moisture, has seen widespread use. Since its invention, usage in China alone has increased from 6,000 tons, covering 117,000 hectares in 1982, to 1.5 million tons, covering 18.4 million hectares by 2016 (Meng et al., 2020). Globally, the mulch market was valued at USD 3.5 billion in 2020 and is projected to reach USD 5.1 billion by 2027 (Mansoor et al., 2022). Most mulches are made of low-density polyethylene (LDPE) (Malinconico et al., 2008) and can degrade into tiny particles, contaminating the soil (Brodhagen et al., 2017). Shade nets and plastic greenhouse covers are also widely used, with an estimated 40,000 km2 of European farmland covered by plastic film (Maraveas, 2020). Additionally, polyvinyl chloride (PVC) and high-density polyethylene (HDPE) particles from irrigation pipes and hoses can enter the soil (Ding et al., 2020). For instance, a study in Turkey found that 13% of microplastics in 10 soil samples originated from disposable drip irrigation pipes (Gündoğdu et al., 2022).

On the other hand, agricultural inputs contribute to MNP accumulation in soil. Compost, an alternative to inorganic fertilizers, contains plastic materials due to ineffective waste separation before composting, making it a source of MPs (Henseler et al., 2022). Compost screening only removes plastic particles up to a certain size. A study in Zhejiang Province, China, found an average of approximately 2,400 MP items per kilogram of dry weight in compost derived from rural domestic waste (RDW) (Gui et al., 2021). Additionally, controlled-release fertilizers (CRFs) are favored for improving nutrient use efficiency, accounting for 16.8% of the global fertilizer market, valued at approximately USD 12.67 billion in 2021 (Controlled Release Fertilizer Market Size and Share Analysis, 2016–2028). However, polymer-based coatings used in CRFs can degrade into MPs, posing risks to soil (Kumar et al., 2020). The encapsulation of agrochemicals using MNP carriers, often petroleum-derived polymers like polyurea and polyolefins, has been explored for the prolonged release of active ingredients (Machado et al., 2022). Moreover, tyre abrasion is another significant source of MPs, with particles primarily composed of synthetic polymers that are solid, insoluble in water, and fall within the typical microplastic size range (Leifheit et al., 2022). Sommer et al. (2018) reported that tyre wear contributes to approximately 30% of MP pollution in rivers, lakes, and seas. While its impact may be minimal for smallholder farms, it is more pronounced on large-scale farms with frequent vehicle use. However, the risk from contaminated water and environmental exposure remains significant in all scenarios.

The effects of these primary and secondary factors are intensified by several environmental influences. Wind and surface runoff, in particular, contribute to the distribution of MNPs. Plastic particles from various sources, such as cosmetics and textiles, can be carried by wind over long distances and deposited onto soil through atmospheric deposition (Radford et al., 2023). Rainfall generates runoff that transports MPs from soil surfaces (Duis & Coors, 2016), leading to direct or indirect contamination of agricultural soil. These factors and direct and indirect sources contribute to the accumulation of MNPs in the soil.

## MNPs impact on soil physicochemical properties

Research studies extensively discuss the accumulation of MNPs in agricultural soil and their behavior in the soil environment. According to these findings, once MNPs adhere to soil colloids, they induce changes in several soil properties, including physical and chemical characteristics (Galahitigama et al., 2024). These alterations have significant implications for plant health and soil fauna activities. For instance, the introduction of MPs into soil reduces soil bulk density, a key physical characteristic that influences soil permeability (Liang et al., 2021; Shah et al., 2017). Additionally, this phenomenon can lead to further complications, such as impaired soil water storage capacity, altered microbial activity, pH imbalances, modified soil porosity, and disrupted nutrient transfer (Liu et al., 2018). MNPs, having a lower density than soil minerals, can disrupt soil aggregation (de Souza Machado et al., 2018; Kim et al., 2021). Changes in soil properties due to MNP accumulation also affect the soil’s water-holding capacity (WHC). The decrease in WHC can be attributed to crack formation, increased water evaporation, and the hydrophobic nature of MP surfaces (de Souza Machado et al., 2019; Sajjad et al., 2022; Wan et al., 2019).

Furthermore, MNP contamination can impact soil temperature by either increasing or stabilizing it (Snyder et al., 2015). Amare and Desta (2021) observed that soil contamination with black polythene enhanced the absorption of solar radiation while reducing its reflection. The altered aggregate size caused by MNP contamination leads to changes in the soil’s hydraulic conductivity. Qi et al. (2020a) also reported an immediate decrease in soil hydraulic conductivity following contamination with LDPE-MPs. In contrast, Zhang et al., (2019a, 2019b) found that soil MP contamination increased hydraulic conductivity. Thus, the specific type of MNPs is crucial in influencing these physical characteristics. However, soil texture remains unaffected by MP contamination (Lehmann et al., 2019).

Incorporating MNPs into the soil can significantly alter its chemical characteristics. For example, polyethylene has been found to decrease soil pH (Wang et al., 2020), whereas polystyrene has shown no significant effect (Boots et al., 2019). The decrease in soil pH is attributed to the release of lactic acid from aliphatic polyesters through mineralization (Wang et al., 2020). Another contributing factor is the alteration of soil microbes that release hydrogen cations. Compounds such as phenanthrene, found in MNPs, can limit the abundance of ammonia-oxidizing bacteria, thereby reducing hydrogen cation release (Yi et al., 2020). Furthermore, MP contamination affects soil organic matter content, as a substantial amount of acidic soil organic matter can lower soil pH (Rezaei et al., 2019). Zhao et al. (2021) suggest that MPs can also enhance soil pH, depending on the plastic's shape, type, and exposure time.

Microplastic contamination may also decrease soil sorption and cation exchange capacities, limiting the attachment of molecules (Strawn, 2021). This reduction in soil sorption can facilitate the adsorption of heavy metals such as cadmium (Chen et al., 2021), which can harm plants and pose environmental and health risks. Different types of MPs can impact the soil’s carbon/nitrogen (C: N) ratio differently. For instance, polyamide microplastics can reduce this ratio, while LDPE-MPs can increase it (Qi et al., 2020b). Polyamides contain nitrogen, which can be released into the soil environment in significant amounts, influencing the C: N ratio (Souza Machado et al., 2019). Conversely, most MPs contain carbon compounds that can be gradually released over time, regardless of their biodegradability (Qi et al., 2020a, c). Additionally, MNP contamination can affect nutrient cycling due to changes in the C: N ratio (Khalid et al., 2020). MNPs also significantly impact soil nutrient dynamics, often leading to imbalances in carbon and nitrogen levels (Torres et al., 2021). This imbalance primarily arises from observed changes in carbon and nitrogen mineralization processes. Soil contamination with MNPs can stimulate and activate soil enzymes such as catalase and urease, which are influenced by soil moisture levels (Huang et al., 2019). These enzymes, primarily produced by microbes, play a crucial role in nutrient cycling within the soil (Ashraf et al., 2021). The influence of MNPs on soil microbial functions is discussed in Sect. 9 of this paper.

## Uptake and translocation of MNPs in vegetable crops

**Fig. 7:**
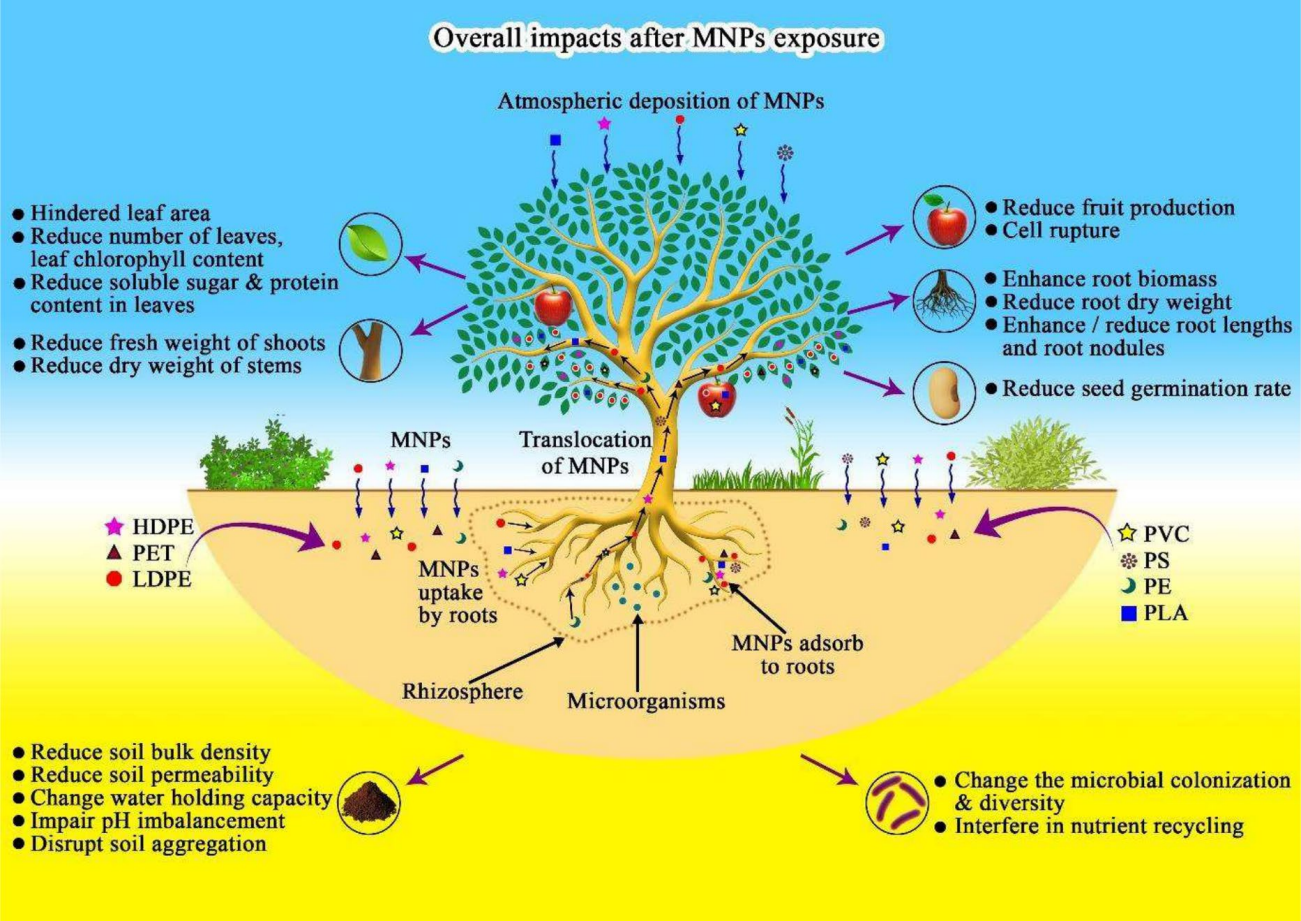
The overall effect of MNPs on vegetable crop growth and development. The schematic diagram illustrates how commonly encountered MNP particles are absorbed into vegetable plants via roots and leaves, then translocated and bioaccumulated in various plant parts. This accumulation of MNPs leads to phytotoxic effects that inhibit plant growth. Additionally, MNPs in the soil alter its properties and disrupt microbial activities in the rhizosphere

Many studies have assessed the entry and transport of MNPs into and within plants. MNPs are primarily taken up by root hairs, transported to the stem, and then translocated to the leaves and fruits. Additionally, entry can occur through foliar application or atmospheric deposition, which allows transfer to other plant parts through stomata (Bosker et al., 2019; Galahitigama et al., 2024; Fig. 7). In this context, plastic particles are absorbed into roots by endocytosis. However, the absorption site into vascular plant tissues varies depending on the crop type (Yin et al., 2021). The uptake and translocation of plastic particles in plants depend on their size, shape, and surface charge (Azeem et al., 2021). Factors such as plant type, condition, and age also play a role (Roy et al., 2023). Nanoplastics (NPs) are more likely to penetrate plant cell walls than MPs due to physical barriers that prevent larger particles from entering. However, MPs can still be absorbed onto plant root and seed surfaces under certain conditions (Azeem et al., 2021; Roy et al., 2023). The root serves as the first barrier for MNPs entering plants. Root cap cells release mucilage to aid in root penetration into the soil. Studies have shown that MNPs can become trapped in root cap mucilage, which performs protective functions (Roy et al., 2023). A study on lettuce demonstrated that 18 μm PVC powder can be absorbed and transported to the leaves, whereas 150 μm powder cannot (Li et al., 2020a, 2020b).

Even though it is commonly believed that larger MPs cannot enter plant tissues, studies have shown that the upper size limit for plastic particle absorption varies. The crack-entry mode may facilitate the entry of larger particles into plants (Li et al., 2020a, 2020b). Such openings can occur when physical barriers, such as the Casparian band in roots, are incomplete due to ageing, herbivore damage, mechanical injury, or secondary root initiation. Sub-micrometer and micrometer-sized MPs can accumulate and be transported within plants through this crack-entry mode (Li et al., 2020a, 2020b). MPs adsorb onto the root surface and move through the root system with water and nutrients into the shoots, utilizing the transpiration pull of the vascular bundle (Igalavithana et al., 2022). After entry, these particles travel from the root surface through the cortex toward the vascular bundle via apoplastic transport, with the transpiration pull serving as the main driving force (Wu et al., 2021a, 2021b).

Some studies have examined the influence of MP shape on their uptake and translocation within plants. For example, Van den Berg et al. (2020) found that wheat roots more easily absorbed spherical MPs than fibrous and flaky MPs. In this case, spherical MPs accumulated more in root tissues than in shoot tissues, whereas fibrous and flaky MPs exhibited the opposite pattern. Another study by Sadler et al. (2019) on tomato plants found that the presence and accumulation of MPs in plant tissues varied depending on their shape, size, and concentration. Despite this evidence, no clear correlation between MP shape and plant uptake has been established.

## Impact of MNPs on oxidative responses

Microplastic uptake by plants leads to oxidative stress by damaging cellular components (Giorgetti et al., 2020). ROS, or oxygen-containing molecules generated as by-products of cellular metabolism, can harm DNA, proteins, and lipids in plant cells (Qiu et al., 2022). While ROS are natural by-products of plant metabolism, MP uptake stress can also induce their production (Ekner-Grzyb et al., 2022). The primary forms of ROS include superoxide anion (O₂·), hydrogen peroxide (H₂O₂), hydroxyl radical (·OH), and singlet oxygen (^1^O₂), each differing in origin, lifespan, reactivity, and biological effects. Additional ROS, such as peroxy, alkoxy, hydro-peroxy radicals, peroxynitrite, ozone, and hypochlorous acid, may also be involved (Demidchik, 2015). Superoxide anion is a primary ROS produced in vegetable plants in response to MP exposure. It can interact with other ROS and biomolecules, leading to lipid peroxidation, protein peroxidation, and DNA damage. Conversely, the hydroxyl radical is the most reactive ROS, formed via the Fenton reaction involving H₂O₂ and ferrous ions. It can severely damage cellular components and is often used as a marker of oxidative stress (Ekner-Grzyb et al., 2022). In this regard, Lian et al., (2021a, 2021b) identified key indicators of oxidative stress in lettuce leaves exposed to polystyrene nanoplastics (PS-NPs) and reported that total antioxidant capacity decreased by 12.4%–26%. Similarly, Gao et al. (2019) reported that H₂O₂ content in leaves and roots increased by 25.26% and 25.31%, respectively. In contrast, Shi et al. (2023) highlighted a significant reduction in H₂O₂ content in tomato leaves and roots treated with polypropylene microplastics (PP-MPs), showing decreases of 87.29% and 62.13%, respectively, compared to the control.

Numerous studies show that even small NP exposure can cause ROS accumulation, triggering plant oxidative bursts, damaging cells, and disrupting metabolism (Senavirathna et al., 2021). This reduces photosynthetic pigments, increases lipid peroxidation, and cell wall damage (Kang et al., 2023). Furthermore, Cui et al. (2022) found that MPs in soil induced oxidative damage in cherry radish roots, inhibiting growth, as evidenced by increased MDA content. Under oxidative stress, plants activate antioxidative defense mechanisms, including peroxidases, catalases, superoxide dismutase, and nonenzymatic antioxidants (Roy et al., 2023). Exposure to PS-MPs and phenanthrene induced oxidative stress in soybean plants, as indicated by significantly increased ROS and MDA contents in the roots. Additionally, catalase activity increased in soybean roots exposed to MPs and phenanthrene (Xu et al., 2021). Zhang et al., (2023) reported that low and high concentrations of polyethylene microplastics (PE-MPs) significantly increased peroxidase content by 67.95% and 49.40%, respectively. However, the same study further described the inhibition of CAT activity in plants by 48.77% and 25.58%.

## MNPs on physio-morphology and pigmentation

The presence of MNPs in the terrestrial environment causes physiological, morphological, and ecological changes in crops. MNPs interfere with various physiological processes, including seed germination, photosynthesis, respiration, transpiration, nutrient movement, and stomatal functions (Amelba et al., 2022). MPs are more likely to delay or prevent seed germination, leading to slower plant development (Lian et al., 2021a, 2021b). This may be due to MPs accumulating in seed pores, hindering germination, growth, and reproduction (Amelba et al., 2022). Soil-accumulated MPs can also adhere to the surface of germinating seeds, physically blocking seed capsule pores and preventing the entry of water and nutrients during germination, which is crucial for root system development (Oliveri Conti et al., 2020).

MNP exposure to roots has also shown negative impacts on above-ground plant parts. According to Lian et al. (2021a), the dry weight, plant height, and leaf area of lettuce significantly decreased by 14.3%–27.3%, 24.2%–27.3%, and 12.7%–19.2%, respectively, due to foliar exposure to PS-NPs. Additionally, Rychter et al. (2019) reported a negative impact of poly-2-ethyl-2-oxazoline (PEtOx) on radishes, inhibiting germination by 19% at a contamination level of 1000 mg kg⁻^1^ of soil. On the other hand, Meng et al. (2021) found that the leaf area of common beans treated with 1% LDPE-MPs was larger (724 cm^2^) than that of plants without MP treatment (626 cm^2^).

Photosynthesis is vital for ecosystem sustainability, and disruptions can negatively impact ecosystems and human societies. The process involves light absorption, electron transport, phosphorylation, and carbon assimilation, with pigments playing a crucial role in absorbing light (Li et al., 2021). The influence of MNPs on photosynthetic pigments varies depending on plant species and the type, size, and concentration of MNPs (Li et al., 2021; Pignattelli et al., 2020). According to Lian et al., (2021a, 2021b), chlorophyll a, chlorophyll b, and carotenoid levels were reduced by 9.1%, 8.7%, and 12.5%, respectively, under 1 mg L⁻^1^ PS-NP exposure. Additionally, total chlorophyll content in radish was significantly reduced in the presence of branched polyethyleneimine (BPEI) (Rychter et al., 2019). In this context, the total chlorophyll content in radish seedlings decreased by approximately 19% at the highest tested concentration of BPEI compared to the control. Dong et al. (2021) also observed a reduced chlorophyll content in lettuce after plants were treated with dibutyl phthalate (DBP) and polystyrene, individually or in combination. This reduction could be due to intracellular ROS caused by contaminants, which affect chloroplasts and inhibit chlorophyll synthesis. Additionally, DBP in lettuce leaves might interfere with chlorophyll function by altering the structure of water-soluble chlorophyll protein (Dong et al., 2021). However, contradictory results were reported by Pignattelli et al. (2020), who found that chlorophyll content in *Lepidium sativum* was unaffected by MNP exposure.

Root activity, a key indicator of plant absorption, influences root development, metabolism, and nutrient uptake, ultimately affecting aboveground growth (Dong et al., 2021). A hydroponic experiment demonstrated a reduction in root activity by 3.81%–11.3% with small-sized NPs and 3.20%–10.7% with large-sized NPs after lettuce was exposed to high concentrations of polystyrene (Dong et al., 2021). Gao et al., (2021a, 2021b) further reported reductions in total root length, total root surface area, average root diameter, and the number of root hairs in purple lettuce and green lettuce by 7.37% (7.12%), 17.98% (15.03%), 15.61% (10.26%), and 13.96% (11.59%), respectively, after polyethylene treatment. Furthermore, Bouaicha et al. (2022) observed that the average root diameter in cucumber plants was significantly reduced by 14%, 12%, and 10% for low, medium, and high PE-MP concentrations, respectively. Similarly, Shi et al. (2023) reported that MPs impacted root biomass in tomato plants, showing an apparent decline under small-sized PS (5.23 µm) and small-sized PE (11.15 µm) treatments compared to the control. Additionally, root performance varies depending on the type of MNPs. For example, root biomass increased with polyester (PES) and polystyrene (PS) treatments, whereas high-density polyethylene (HDPE), polyethylene terephthalate (PET), and polypropylene (PP) had a lesser impact on root performance (De Souza Machado et al., 2019).

## Impact of MNPs on metabolism

According to research on plant metabolomics, MNPs can alter a range of primary and secondary metabolites, including carbohydrates, lipids, proteins, amino acids, organic acids, vitamins, and phytohormones. Metabolomics, which provides insights into chemical composition and changes in metabolic processes, has been used to study alterations in metabolites and metabolic pathways in response to environmental stresses and pollutants in plants (Wu et al., 2020). Most research articles focus on the effects of MNPs on metabolomics in areas such as organic acid, fatty acid, carbohydrate, and amino acid metabolism (Wu et al., 2020). For instance, Zeb et al. (2022) found that adding 0.2% microfibers (MFs) significantly altered 17 metabolites in lettuce leaves, including amino acids, carboxylic acids, fatty acids, sugars, and sugar alcohols.

MNPs affect the metabolic pathways of terrestrial plants, including crops, by impairing nutrient absorption, energy production, biosynthesis, and antioxidant defences (Roy et al., 2023). Among the various metabolic compounds in plants, most researchers have focused on amino acid behaviour following MNP exposure. Amino acids are essential for protein synthesis, managing deficiencies, responding to environmental challenges, and overall growth. According to Lian et al. (2021a), PS-NP-treated lettuce exhibited significant reductions in the levels of essential amino acids such as lysine, tryptophan, threonine, isoleucine, leucine, valine, and histidine. Additionally, levels of non-essential amino acids were also altered, including proline, tyrosine, serine, aspartate, arginine, asparagine, and ornithine. Specific interactions and bonds, such as π-π and cation-π interactions, that form between amino acids and MNPs result in different adsorption behaviors of multiple amino acids by MNPs, which may explain the uneven concentration of amino acids in plants (Jiang et al., 2022). High levels of oxidative stress caused by MNPs damage secondary metabolites, including amino acids and lipids (Zantis et al., 2023). For example, vegetable crops exposed to PS-MPs exhibit increased ROS production, leading to oxidative damage (Iqbal et al., 2023). ROS oxidizes cysteine residues and forms disulfide bridges, affecting protein structure and function (Ekner-Grzyb et al., 2022).

Additionally, MPs alter gene expression by interfering with the alanine, aspartate, glutamate, carbohydrate, and amino acid pathways (Iqbal et al., 2023). For instance, cucumbers' soluble sugar, vitamin C, and osmoregulation chemicals (such as soluble protein and proline) varied when exposed to PS-NPs (Li et al., 2021). Similarly, PS concentration in carrots decreased soluble sugar, α-carotene, and β-carotene levels (Dong et al., 2022a). Another study conducted by Lian et al. (2021a) found that PS-NPs deteriorated the nutritional quality of lettuce leaves by reducing the concentrations of micronutrients, including Fe, Zn, and essential amino acids. Moreover, Gao et al., (2021a, 2021b) showed that the DBP + PE treatment reduced the amount of soluble protein and sugar in purple (green) lettuce leaves from 4.40% to 13.99% (4.02% to 11.48%) and from 1.73% to 7.94% (1.34% to 5.23%), respectively. A hydroponic study conducted by Dong et al. (2021) reported that small-size PS (100–1000 nm) and large-size PS (> 10,000 nm) increased root acetylsalicylic acid (ASA) and reduced glutathione (GSH) by 12.3%–29.5% (11.5%–40.0%) and 10.7%–26.0% (9.8%–21.1%), respectively. Table 1 summarizes the impact of MNPs on the growth and development of vegetable plants.

**Table 1:** Impact of numerous MNPs on the growth and development of several vegetable crops

Crop	Polymer type	Polymer concentration	Polymer size/diameter	Growing condition	Major findings	References
Lettuce (*Lactuca sativa*)	PS	0.1–1 mgL^−1^	0.093 µm	Soil	Reduced micronutrients and essential amino acids content Decreased the dry weight, height, and leaf area of lettuce compared with the control	Lian et al., 2021a, 2021b
	PS	1,2,3,4,10,20 mgL^−1^	0.1–5 µm	Hydroponic	Decreased soluble protein and sugar content	Dong et al., 2021
					Increased H_2_O_2_ and MDA content in roots	
	PE	0.25, 0.5, 1 mgmL^−1^	23 μm	Hydroponic	Reduced soluble protein and sugar content in lettuce leaves, increased vitamin C content	Gao et al., 2021a
					Increased reticulum vesicles and cell rupturing	
	PS	0.25, 0.5, 1 mgmL^−1^	0.1–1.0 μm	Hydroponic	Damaged cell membrane and wall Hindered lettuce biomass	
	PE	0.25, 0.5, 1.0 mgmL-1	23 μm	Hydroponic	Decreased photosynthetic rate, stomatal conductance, transient transpiration rate, chlorophyll content including chlorophyll a, chlorophyll b, and activity of Rubisco	Gao et al., 2019
					Increased intercellular CO_2_ concentration	
Common bean (*Phaseolus vulgaris*)	LDPE	0.5, 1.0, 1.5, 2.0, 2.5 w/w (dry soil basis)	53–1000 μm	Soil	Increased root nodules, length and leaf area, Decreased chlorophyll content	Meng et al., 2021
Broad bean (*Vicia faba*)	PS	10, 50, 100 mgL^−1^	0.1 −5.0 μm	Hydroponic	Induced higher genotoxic and oxidative damage Decreased biomass and CAT enzyme activity	Jiang et al., 2019
Onion (*Allium cepa* L.)	PS	0.01, 0.1, 1 gL^−1^	0.05 μm	Hydroponic	Cytotoxicity and genotoxicity symptoms were observed Decreased mitotic index	Giorgetti et al., 2020
	PS	25, 50, 100, 200, 400 mgL^−1^	0.1 µm	Hydroponic	Reduced root length, mitotic index, induced chromosomal and nuclear aberrations Abridged mitotic index	Maity et al., 2020
Chinese cabbage (*Brassica chinensis* L.)	HDPE, GPPS	2.5, 5, 10, 20 gKg^−1^	25–850 µm	Soil	Reduced soluble sugar concentration, concentration of leaf chlorophyll, and fresh weight	Yang et al., 2021
Spring onion (*Allium festulous*)	PE, PET, PP, PS	0.2–2.0 w/w	8–500 μm	Soil	Increased root biomass but decreased root, leaf and bulb dry biomass	de Souza Machado et al., 2019
Radish (*Raphanus sativus*)	PEIs	100, 250, 500, 750, 1000 mgKg^−1^	N/A	Soil	Increased nitrogen content in green parts	Rychter et al., 2019
Garden cress (*Lepidium sativum*)	PP, PE, PVC	0.02 w/w	125 μm	Soil	Reduced rate of germination, leaf number, and biomass Decreased seed germination	Pignattell et al., 2020
Tomato (*Lycopersicon esculentum*)	PET, PVC	1.29–1.40 g cm^−3^ and 1.30–1.58 g cm^−3^	> 1.2 μm	Soil	Increased growth but decreased fruit production Higher shoot and root biomass	Hernández-Arenaset al., 2021
	PS	0.1, 1 mgL^−1^	5.23–17.21 μm	Hydroponic	Inhibited root and shoot growth Decreased the root mass	Shi et al., 2023
	PE	0, 10, 100, 1000 mgL^−1^	0.79 – 4.99 μm	Distill water	Inhibited germination and root growth Induced shoot growth	Bouaicha et al., 2022
Water spinach (*Ipomoea aquatica* Forsk)	PS	0.5 −10 mgL^−1^	0.08 μm	Hydroponic	Declined root growth and shoot growth	Song et al., 2023
Cucumber (*Cucumis sativus L.)*	PE, PLA	200 mgL^−1^	13, 48, and 500 μm	Hydroponic	Inhibit the root growth and shoot growth. Inhibited the photosynthesis of seedlings and caused lipid peroxidation Enhanced the Cr accumulation in cucumber root	Zhang et al., 2023
Sweet potato (*Ipomoea batatas L.)*	PVC	100–200 mgL^−1^	6.5 μm	Hydroponic	No change in root growth and shoot growth. However, PVC- MPs enhanced Cr (VI) accumulation and toxicity	Khan et al., 2023

GPPS- General purpose polystyrene, HDPE- High-density polyethylene, LDPE-MPs-Low-density polyethylene microplastics, PEIs –Polyethylenimines, PE-Polythene, PET-Polyethylene terephthalate, PLA-Polylactic acid, PP-Polypropylene, PS-NPs-Polystyrene nanoparticles, PS-Polystyrene, PVC- Polyvinyl chloride, μm-micrometer, mgL^−1^-mg per litre, gKg^−1^-g per kilograms, w/w-weight by weight, gcm^−3^-g per cubic centimeter, mgmL^−1^- milligrams per milliliter

## Effect of MNPs on transcriptomic gene profiles

The molecular basis of MP-induced responses has been found to be significant. MNPs upregulate stress-responsive genes, helping plants cope with adverse conditions (Liu et al., 2022; Zhang et al., 2021). Studies show that MNPs activate plant defense mechanisms by upregulating genes involved in the production of secondary metabolites, antioxidants, and other defense compounds (Wang et al., 2022a, 2022b; Martin et al., 2023; Zhang et al., 2023; Li et al., 2024). MPs also affect the expression of genes related to nutrient transport and metabolism, impacting plant physiology (Liu et al., 2023a, 2023b). According to Liu et al., (2023a, 2023b), polypropylene and rubber crumbs inhibited nitrogen uptake and slowed vegetative growth in peanuts due to plasma membrane damage in root cells and disruption of nitrogen cycling. Concurrently, MPs alter gene expression related to root growth, morphology, and nutrient uptake (Li et al., 2023a, 2023b; Yang & Gao, 2022).

On the other hand, some epigenetic changes are induced by MP exposure (López de Las Haza et al., 2022; Babele & Bhatia, 2023). Epigenetic modifications affect gene expression without altering DNA sequences. Different plant species exhibit varying transcriptomic responses to MNP exposure. Recent studies have reported transcriptomic responses in horticultural crops exposed to MPs. For instance, lettuce exposed to various concentrations of fluorescence-labelled PS-MPs showed changes in antioxidant enzyme genes, including six CAT-related and four APX-related genes. Notably, an MP concentration of 10 mg L⁻1 significantly altered these gene expressions, indicating a concentration-dependent influence on oxidative stress (Wang et al., 2023).

Another study examined the effects of PE-MPs, specifically blue microbead microplastics (B-MP) and white microbead microplastics (W-MP), on *Zea mays* seedlings cultured in vitro. White microbead microplastics (W-MP) affected the highest number of differentially expressed genes (DEGs), with 16,402 and 12,180 DEGs identified. Gene ontology (GO) enrichment linked these DEGs to functions such as "heme binding," carbohydrate transport, "phenylalanine ammonia-lyase activity," "peroxidase activity," and "oxidoreductase activity." Seven key genes were significantly influenced, including proton myo-inositol (Gene ID 100193474), sorbitol transporter (Gene ID 100281055), and putative polyol transporters (Gene IDs 100,273,244, 100,279,532, 100,381,931, 103,647,781, and 100,273,244) (Martin et al., 2023). When exposed to MPs, melon (*Cucumis melo* L.), often cultivated using plastic mulch film, exhibits significant transcriptomic changes. A study revealed the upregulation of 16 genes related to plant hormone signal transduction and plant-pathogen interaction and the downregulation of 13 genes related to plant hormone signal transduction, metabolism, and plant-pathogen interaction (Li et al., 2023a, 2023b).

In addition, Zhuang et al. (2023) reported that polystyrene microplastics (PS-MPs) affect photosynthesis and carbon- and nitrogen-metabolism-related genes in cucumbers (*Cucumis sativus* L.). A PS concentration of 5 μm significantly impacted genes essential for NADPH and ATP synthesis in photosynthesis. Additionally, PS at 0.1 μm negatively affected genes involved in phosphoenolpyruvate carboxykinase (PEPCK) and phosphoenolpyruvate carboxylase (PEPC), which are related to CO₂ concentration, as well as nitrate/nitrite transporter (NRT) and nitrate reductase (NR) activity, thereby reducing nitrogen use efficiency in cucumber leaves. Studies addressing the impact of MNPs on the transcriptomes of horticultural crops highlight the need for further research. Existing studies indicate that MNPs alter genes related to oxidative stress, carbohydrate transport, plant hormone signal transduction, photosynthesis, and plant-pathogen interaction. Moreover, MPs combined with heavy metals significantly harm crop growth, and understanding the transcriptomic basis will help clarify the regulatory mechanisms involved. MNP exposure in onion (*Allium cepa* L.) has shown cytotoxic and genotoxic effects in the root meristem. Giorgetti et al. (2020) reported that the mitotic index decreased from 9.3% in control meristems to 6.1% and 5.4% in 0.1 and 1 gL⁻^1^ treatments, corresponding to 34.4% and 41.9% reductions, respectively. Table 2 summarizes the effects of MNPs on the transcriptomic gene profiles of vegetable crops.

**Table 2:** The effects of MNP on transcriptomic gene profiles of vegetable crops

Crop	Type of MNP	Concentrations	Affected gene	Reference
Lettuce (Lactuca sativa)	Fluorescence-labelled PS-MPs	0, 10, 20, 30, 40, 50 mgL^−1^	Antioxidant enzymes-CAT related genes and four APX genes	Wang et al., 2022a, 2022b; Wang et al., 2023
Melon (Cucumis melo L.)	PVC-MP	1, 2, 4, 8,16 gKg^−1^	Up-regulation of 16 genes related to plant hormone signal transduction and plant-pathogen interaction and 13 down-regulated genes related to plant hormone signal transduction, metabolism-related genes and plant-pathogen interaction	Li et al., 2023a, 2023b
Cucumber (Cucumis sativus L.)	PS-MPs	0.1 μM and 5 μM	Genes necessary for the NADPH and ATP synthesis in photosynthetic pathway Negatively affects the genes involved in phosphoenolpyruvate carboxykinase (PEPCK) and phosphoenolpyruvate carboxylase (PEPC) related to CO_2_ concentration inside cells NRT and NR activity diminishing the nitrogen use efficiency in cucumber leaves	Zhuang et al., 2023

PS-MPs-Polystyrene microplastics, B-MP- Blue microbeads microplastics, W-MP-White microbeads microplastics, PVC-MP- Polyvinyl chloride microplastic, CAT- Catalase, APX- Ascorbate peroxidase, NADPH-Nicotinamide adenine dinucleotide phosphate, ATP- Adenosine triphosphate, μM-micromole, mgL^−1^-milligrammes per litre, gKg^−1^-g per kilograms

## Effect of MNPs on horticultural crop rhizosphere

The rhizosphere is a key driver of plant growth. The relationship between the rhizosphere and plant roots is crucial for nutrient uptake and stress tolerance, ultimately influencing crop growth. Microorganisms in the rhizosphere play a vital role in maintaining stable plant-soil interactions (Trivedi et al., 2020). However, most studies indicate that MNPs pose a significant threat to rhizosphere diversity and function, thereby affecting crop growth. In addition to their direct effects, MNPs indirectly influence rhizosphere changes by altering soil pH, structure, and crop interactions.

The impact of MNPs on microbial communities is complex and dynamic, with studies indicating various effects on soil and aquatic ecosystems. MPs provide surfaces for microbial colonization (Hossain et al., 2019a, 2019b). Bacteria and other microorganisms attach to these particles, forming biofilms (Mishra et al., 2022; Zhai et al., 2023). Harrison et al. (2014) found that bacteria in marine sediments colonize LDPE-MPs, creating plastisphere-specific bacterial communities. This colonization may influence microbial diversity, abundance, and activity. Several studies have reported changes in microbial diversity due to MPs (Yuan et al., 2023). These changes depend on the type of plastic, environmental conditions, and microbial taxa (Mughini-Gras et al., 2021). MPs can promote biofilm formation, influencing nutrient dynamics and plastic degradation (He et al., 2022; Sooriyakumar et al., 2022). Huang et al. (2019) demonstrated distinct bacterial assemblages on LDPE-MPs, including plastic-degrading bacteria. Furthermore, Yuan et al. (2023) found that polyethylene and polypropylene MPs affect saline soil microbial communities, with fungal diversity being more impacted than bacterial diversity, thereby enhancing enzyme activities and soil nutrient content. MPs also influence nutrient cycling by affecting microbial activity, providing substrates for microbial growth, and altering nutrient availability and cycling (Tang et al., 2023). Rong et al. (2021) showed that LDPE-MPs in soil increased the abundance of *nifH*, *AOBamoA*, and *nirK* genes involved in nitrogen cycling, impacting soil bacterial networks and functional groups. Thus, MPs significantly affect ecosystem functioning and nutrient availability for plants and other organisms.

Another study reports the effects of two types of MP-polystyrene beads and degradable mulching film (DMF) on rhizosphere changes (Ren et al., 2022). It found a significant increase in the abundance of dominant fungi, such as *Fusarium*, which causes Fusarium blight, and *Alternaria*, in the presence of these MPs, highlighting the selective effect of MPs on crop pathogens (Ren et al., 2022). MNPs and phthalate esters (PAEs) also significantly affect the abundance of crop endophytes, such as *Proteobacteria*. *Proteobacteria* are crucial for symbiotic relationships in microbial communities and enhance root biomass by synthesizing the superoxide dismutase enzyme (Gao et al., 2024; Table 3).

**Table 3:** Numerous MNP's effects on rhizosphere structure and functions

Crop	Type of MNP	Alternations in rhizosphere structure and function	Reference
N/A	MNPs and PAEs	*Proteobacteria*	Gao et al., 2024
Spinach (*Ipomoea aquatica*)	PE-MPs	Increased *Protobacteria* and *Gamma-proteobacteria,* whereas *Firmicutes* and *Actinobacteria* decreased For the fungal community, *Glomeromycota* and *Ascomycota* were the dominant phyla, and Glomer Omycetes and Paraglomeromycetes were the dominant classes among the root endophytes	Yu et al., 2023
Pakchoi (*Brassica chinensis* L.)	PE	Negatively affects the relative abundance of bacterial phyla *(Actinobacteria, Acidobacteria, Gemmatimonadetes, Bacteroidetes, Verrucomicrobia, Nitro spirae*, and *Candidatus_Rokubacteria*) and the fungal phylum (*Ascomycota*)	Han et al., 2023

*MNPs-*Micro and nano plastics, *PAEs*-phthalate esters, *PS*-Polystyrene, *PE-MPs*-Polyethylene microplastic, *PE*- Polyethylene, *PLA*- Polylactic acid

Another critical aspect is the influence of MNPs on the horizontal gene transfer (HGT) of antibiotic resistance genes. Polystyrene (PS) particles of varying sizes have significantly affected HGT in rhizosphere bacteria. PS-MPs promote the acquisition of antibiotic-resistance genes (ARGs) by pathogenic and nitrifying bacteria (Zhao et al., 2023).

Yu et al. (2023) studied the effect of polyethylene microplastics (PE-MPs) on the rhizosphere microbial and root endophyte community diversity of spinach (*Ipomoea aquatica*). To demonstrate this effect, they used PE particles of 0.5 μm and 1 μm at concentrations of 0.5% and 1% (w/w) in a pot experiment. Another study investigated the impact of various MPs, including polyethylene, polystyrene, polylactic acid, and a weathered microplastic mixture, on microbial reductive dehalogenation at both strain and community levels. These MPs increased dehalogenation in *Dehalococcoides* by 10–217%. However, PS-NPs inhibited dehalogenation due to increased ROS production. This study confirmed that variations in *organohalide-respiring bacteria* (OHRB) growth and *reductive dehalogenase* (RDase) gene transcription were responsible for this effect (Liu et al., 2023a, 2023b). These findings highlight the need for greater attention to the fate of organohalides with increasing MP pollution, as organohalides play a significant role in crop growth (Berry et al., 1975).

## Remediation strategies to mitigate MNPs toxicity

Significant MNP buildup in agricultural soils is recognized as harmful to the ecosystem. However, there is a need to standardize and develop specific prevention and control measures for MP pollution in agroecosystems. The main preventive and control measures that should be implemented include enacting laws, promoting the use of biodegradable plastics, and regulating the recycling and disposal of plastic waste.

Farmers use plastic mulches due to their flexibility, ease of mechanical application, and cost-effectiveness compared to natural mulches (Miles et al., 2017). Plastic mulches substantially benefit crop production by controlling weeds, managing certain plant diseases, conserving soil, and maintaining microclimates (Sintim et al., 2019). However, they have a significant environmental impact by introducing MNPs into the soil (Rout et al., 2022). Most plastic mulches persist beyond crop cycles, further compounding their environmental footprint (Sajjad et al., 2022). To promote sustainability, transitioning from plastic to biodegradable alternatives is recommended. Organic materials such as crop residues, tree leaves, rice straw, husks, wood dust, and water hyacinths are advocated for mulching to mitigate these impacts (Rout et al., 2022). Biodegradable plastic mulches, including polyhydroxybutyrate (PHB), polybutylene succinate, polycaprolactone (PCL), polyhydroxyalkanoate (PHA), polybutylene adipate-co-terephthalate (PBAT), polylactic acid (PLA), polyglycolide, and starch-based blends, offer effective alternatives for crop production (Miles et al., 2017). Ensuring that these biodegradable mulches are entirely bio-based is essential to guarantee their degradation without negatively affecting soil health.

In addition, research studies indicate that biodegradable plastic mulches enhance raspberry plant growth and yield (Zong et al., 2021), while their effects on earthworms vary, necessitating further investigation (Qi et al., 2018). Plastic-coated fertilizers are another significant source of MNP contamination in agricultural lands (Sajjad et al., 2022). During irrigation seasons, estuarine waters exhibit increased levels of MPs, with 90% attributable to MNPs from fertilizer coatings (Lwanga et al., 2022). To mitigate this issue, natural polymers can replace plastic in polymer-coated fertilizers (Roy et al., 2023). Furthermore, the application of sewage or treated wastewater is a significant route for introducing MNPs into the soil (Wang et al., 2022a, 2022b). Strict regulations would help limit the use of potentially harmful wastewater in agriculture. Processing steps such as drying, pasteurization, and composting should reduce MNPs in treated sewage. International monitoring programs, research into ecological impacts, and awareness campaigns among farmers and wastewater treatment operators are crucial for addressing this issue (Nizzetto et al., 2016). Advanced technologies such as membrane processes, advanced oxidation, electrocoagulation, and nanotechnologies aid in removing MNPs from wastewater and soil (Rout et al., 2022). Phytoremediation, which involves using plants to remove or degrade pollutants like MNPs from soil, is another promising approach (Zhang et al., 2019a, 2019b). Some plants, such as soybean and wheat, effectively absorb and accumulate MNPs, thereby reducing soil concentrations (Zong et al., 2021).

Biochar or surfactant amendments effectively immobilize pollutants in soil (Sun et al., 2018). Biochar enhances soil properties and reduces the bioavailability of heavy metals and organic contaminants (Bousdra et al., 2023). Encapsulated enzyme treatments, using enzymes like PETase and MHETase extracted from *Ideonella sakaiensis* and encapsulated in lignin shells, show promise in MNP remediation (Roy et al., 2023). Biological degradation through aerobic or anaerobic biodegradation is also a viable technology for MNP remediation in soil (Zeenat et al., 2021). For instance, bacteria such as *Bacillus* sp. and fungi from specific ecosystems have been found to degrade various types of MPs (Li et al., 2023a, 2023b). Implementing stringent global regulations is essential for effective MNP management. Policies promoting the responsible production, usage, and recycling of MNPs, supported by robust monitoring, are urgently needed to mitigate MNP accumulation in agricultural soil and ecosystems (Galahitigama et al., 2024). Raising public awareness and promoting recycling and proper disposal of plastic waste is critical in mitigating the environmental impact of MNPs.

## Conclusion

Micro and nanoplastic pollution has become a global environmental issue over the last decade. Humans are primarily affected through the ingestion of MNP-contaminated vegetables. While there is a growing trend to explore the effects of MNPs on vegetable production, studies are currently limited to a few countries and focus mainly on a small number of crops, such as lettuce, tomato, cucumber, and common beans. Consequently, the impact of MNPs on other crops remains unclear. MNPs are primarily absorbed through plant roots; however, the amount of MNP uptake by vegetable plants varies depending on the type and concentration of MNPs and the crop variety. Additionally, current research indicates that MNPs directly affect crop growth and development by disrupting photosynthesis, nutrient absorption, and translocation processes, as well as causing metabolic changes at the cellular level. These metabolic changes result from MNP-induced genetic alterations.

Furthermore, transcriptome profile analysis reveals that MNPs upregulate stress-responsive genes, affecting the expression of genes related to nutrient transport and metabolism, antioxidant enzymes, plant hormone signal transduction, and plant-pathogen interactions. Accumulated MNPs in soil alter the physicochemical properties of the rhizosphere and microbial activities, indirectly affecting vegetable plant growth. Specifically, microbial function and structure changes in rhizosphere soil have been observed after MNP exposure. Several control strategies can be employed to mitigate MNP exposure in vegetable plants, including sustainable agronomic practices, novel technological advancements, implementing rules and regulations, and increasing public awareness.

## Data Availability

No datasets were generated or analysed during the current study.

## Abbreviations

µm: Micrometer
AOBamoA: Ammonia monooxygenase subunit –A in ammonia –oxidizing bacteria
APX: Ascorbate peroxidase
ARGs: Antibiotic Resistance Genes
ATP: Adinosine triphosphate
B-MP: Blue microbeads microplastics
BPEI: Branched Polyethylenimine
CAT: Catalase
Ci: Intercellular CO_2_ concentration
CO_2_: Carbondixoxide
CRF: Controlled-release fertilizers
DBP: Dibutyl phthalate
DCD: Dicyanidamide
DEGs: Differentially Expressed Genes
DMF: Degradable Mulching Film
DNA: Deoxyribonucleic acid
ETR: Electron Transport Rate
GB: Glycine betaine
GPPS: General Purpose Polystyrene
Gs: Stomatal conductance
HDPE: High density Polyethylene
HGT: Horizontal gene transfer
kg: Kilogram
LDPE-MPs: Low-Density Polyethylene
LSPS: Large-size Polystyrene
MC: Manure Control
MDA: Malondialdehyde
mg: Miligram
mgL^−^^1^: Miligrams per litre
MHETase: Mono-(2-hydroxyethyl) terephthalate
MNPs: Micro nano particles
MPs: Microplastics
NADPH: Nicotinamide adenine dinucleotide dinucleotide phosphate
nifH: Nitrogenase
nirK: Nitrite Reductase Genes
NPs: Nanoplastics
NR: Nitrate reductase
NRT: Nitrate/Nitrite transporter
NUE: Nutrient Use Efficiency
OHRB: Organohalide Respiring Bacteria
OTC: Oxytetracycline
PA: Polyamide
PAE: Phthalate Ester
PBAT: Polybutylene adipate-co-terephthalate
PCL: Polycaprolactone
PE: Polythene
PEHD: Polyethylene High Density
PEIs: Polyethylenimines
PEPC: Phosphoenolpyruvate Carboxylase
PEPCK: Phosphoenolpyruvate Carboxykinase
PES: Polyester
PET: Polyethylene terephthalate
PET: Polyethylene terephthalate
PEtOx: Poly (2-ethyl-2-oxazoline)
PFE: Pressurized fluid extraction
PHA: Polyhydroxy alkanoate
PHB: Polyhydroxy butyrate
Phe: Phenanthrene
PLA: Polylactic acid
PME: Pectin Methylesterase
Pn: Photosynthetic rate
POD: Peroxidase
PP: Polypropylene
PS: Polystyrene
PS-NPs: Polystyrene Nano Particles
PU: Polyurethane
PVC: Polyvinyl Chloride
RDase: Reductive dehalogenase
RDW: Rural Domestic Waste
RGR: Relative Growth Rate
ROS: Reactive Oxygen Species
RSR: Root shoot ratio
SOD: Superoxide dismutase
SPE: Small sized Polythene
SPS: Small sized Polystyrene
Tr: Transpiration rate
USD: United States Dollars
W-MP: White microbeads microplastics

## Declarations

All authors have read, understood, and have complied as applicable with the statement on "Ethical responsibilities of Authors" as found in the Instructions for Authors.”

### Human and animals participants

No human or animal involvement in the study.
